# Periostitis of the Upper Jaw, in Connection with the Habit of Biting Coin

**Published:** 1851-04

**Authors:** 


### ARTICLE XX.

***Periostitis of the Upper Jaw, in connection with the Habit of Biting Coin.*** Under the care of Mr. Stanley, St. Bartholomew's Hospital.

It is interesting to observe what serious mischief can be set up in scrofulous constitutions by slight exciting causes, and this circumstance is particularly remarkable as regards affections of bone. Intense inflammation of the periosteum is easily produced, especially in young strumous subjects, by injuries which in others would have passed unnoticed, and the severe inflammation of the investment of the bone is not unfrefrepuently followed by either death of the osseous structure, or by that unhealthy ulceration which has been denominated caries.* These facts are well-known, but the exciting causes vary *ad infinitum.* We have lately become acquainted with one of the latter in a patient of Mr. Stanley, of so unusual a nature that we are induced to offer our readers an outline of the case.

The patient is a lad of fifteen, very short for his age, of fair complexion, slight muscular development, and bearing the marks of small-pox, who was admitted into Darker ward, under the care of Mr. Stanley, March 7, 1850. The boy's teeth are extremely irregular; the first bicuspid is on both sides exactly behind the canine, and the rest form a waving instead of a continuous line. On admission it was evident that severe inflammation had attacked the patient's mouth, and the whole row of teeth on the right side were so loosened that any of them could have easily been extracted with the slightest effort of the finger and thumb. The tongue was thickly coated with a yellowish white and moist fur, and the soft parts composing the right cheek considerably inflamed, as well as the mucous membrane covering the hard palate. The gums were tumefied, tender, of a vivid red, and largely partook of the severe inflammation pervading the whole of the upper part of the mouth on the right side, but the mischief stopped abruptly at the mesian line. The face was swollen and flushed, the symptomatic fever of a violent description, and the pain in the affected side of the mouth extremely acute. The boy earnestly requested that the teeth might be extracted, though it seemed rather a pity to do so, since they were quite free from caries. At first sight the case might have been taken for one of erysipelas, but on close examination the interior of the mouth was found to be the seat of the disease, the exciting cause of which gives great interest to the case.

* Necrosis of the upper or lower jaw is, in most cases, but not invariably, the effect of some local cause acting on the adjacent soft parts, and thence on the periosteum and bone.—[Mr. Stanley on Diseases of the Bone, p. 72.

It appears that the patient gains his living by selling vegetables in the streets, and he had carried on this occupation in perfect health until five days before admission, when he began to give each silver coin he received a hearty bite on the right side of the mouth, to ascertain its genuineness—a caution which he adopted as he had been imposed upon with a counterfeit shilling. He had sold on that day more than a pound's worth of vegetables, and soon afterwards noticed a certain amount of pain in the upper jaw. During the two subsequent days the pain went on increasing, but he still followed his avocation, and used his teeth as heretofore, until two days before admission, when the part became so painful that he was obliged to desist.

Mr. Stanley proceeded to extract two of the teeth; their fangs were found covered with an exceedingly vascular soft periosteum, and a probe being passed into the sockets from which the teeth had been drawn, and also between the remaining teeth and gums, it struck against denuded bone. Mr. Stanley fixed upon the opinion that the whole train of phenomena had their origin in acute inflammation of the periosteum, which might be followed by necrosis of the jaw.

The boy was ordered fomentations, purgatives, and put upon low diet. He had subsequently saline draughts and soothing applications, by which means the inflammation was soon subdued. He went on improving for several weeks; his teeth became a little firmer, but the two empty alveoli did not assume a healthy granulating appearance; they looked as if ulceration and purulent secretion were kept up in them by the structure beneath being in a state of disease.

Nothing of a very remarkable nature took place until the 29th of April, about seven weeks after admission, when, on carefully examining the mouth, Mr. Stanley found that the anterior part of both sides of the upper jaw was in a state of caries, and probably likewise of necrosis. Fistulous openings were discovered above both canine teeth; these were discharging a thin purulent fluid, and a probe introduced into them came in contact with rough bone.

Mr. Stanley gave a very unfavorable prognosis of the case; he conceived that certain portions of the jaw had, in all probability, lost their vitality, and that more or less of them would come away after a little time. It was, in the meanwhile, plain that no therapeutical assistance could be rendered, and that nothing could be done but patiently to await the time when portions of bone would be sufficiently loose to allow of removal. There was evidence, however, that the changes of the osseous structure were proceeding with activity, as the secretion of pus was at times so abundant that the purulent matter was discharged by the nose, *viâ* the posterior nares.

After the patient had been about three months in the hospital, the fistulous apertures on either side of the canine teeth became smaller, but they remained quite pervious, and secreted thin pus. The alveoli whence the teeth had been originally extracted were at the same time covered with a grey film, and showed no disposition to granulate. The patient's general health, however, did not suffer, and Mr. Stanley judged that it would be a great benefit to the boy to leave the hospital, and to show himself now and then as an out-patient. He has been seen several times up to the present date, and it is extremely likely that the process of extrusion and reparation will stretch over a considerable space of time.—*London Lancet.*

